# Multichannel Film Dosimetry for Quality Assurance of Intensity Modulated Radiotherapy Treatment Plans Under 0.35 T Magnetic Field

**DOI:** 10.7759/cureus.7334

**Published:** 2020-03-20

**Authors:** Gorkem Gungor, Latif Korkmaz, Namik Kayalilar, Gokhan Aydin, Bulent Yapici, Teuta Zoto Mustafayev, Banu Atalar, Enis Ozyar

**Affiliations:** 1 Radiation Oncology, Acibadem Maslak Hospital, Istanbul, TUR; 2 Radiation Oncology, Acibadem University School of Medicine, Istanbul, TUR; 3 Radiation Oncology, Acıbadem Hospital, Istanbul, TUR; 4 Radiation Oncology, Acıbadem Mehmet Ali Aydinlar University School of Medicine, Istanbul, TUR

**Keywords:** multichannel film dosimetry, imrt, patient qa, mrigrt, viewray, 0.35 t, ebt3

## Abstract

Purpose

To evaluate the intensity modulated radiotherapy (IMRT) quality assurance (QA) results of the multichannel film dosimetry analysis with single scan method by using Gafchromic™ EBT3 (Ashland Inc., Covington, KY, USA) film under 0.35 T magnetic field.

Methods

Between September 2018 and June 2019, 70 patients were treated with ViewRay MRIdian^®^ (ViewRay Inc., Mountain View, CA) linear accelerator (Linac). Film dosimetry QA plans were generated for all IMRT treatments. Multichannel film dosimetry for red, green and blue (RGB) channels were compared with treatment planning system (TPS) dose maps by gamma evaluation analysis.

Results

The mean gamma passing rates of RGB channels are 97.3% ± 2.26%, 96.0% ± 3.27% and 96.2% ± 3.14% for gamma evaluation with 2% DD/2 mm distance to agreement (DTA), respectively. Moreover, the mean gamma passing rates of RGB channels are 99.7% ± 0.41%, 99.6% ± 0.59% and 99.5% ± 0.67% for gamma evaluation with 3% DD/3 mm DTA, respectively.

Conclusion

The patient specific QA using Gafchromic™ EBT3 film with multichannel film dosimetry seems to be a suitable tool to implement for MR-guided IMRT treatments under 0.35 T magnetic field. Multichannel film dosimetry with Gafchromic™ EBT3 is a consistent QA tool for gamma evaluation of the treatment plans even with 2% DD/2 mm DTA under 0.35 T magnetic field presence.

## Introduction

Magnetic resonance image-guided radiotherapy (MRIGRT) exhibits unique advantages for intensity modulated radiotherapy (IMRT) [1-5]. Online adaptive planning using anatomy of the day and continuous cine mode tracking using magnetic resonance imaging (MRI) give great opportunity to reach precise radiotherapy [1]. ViewRay MRIdian® (ViewRay Inc., Mountain View, CA) is the first clinical MRIGRT linear accelerator (Linac) system with 0.35 T magnetic field at the treatment isocenter [1-4]. Both older Co^60^ radioactive source and 6MV flattening filter-free (FFF) photon energy linear accelerator versions of this system have been successfully used for whole body conventional and stereotactic body radiotherapy (SBRT) [1, 2, 4, 5]. However, consistent and accurate patient specific quality assurance (QA) verification procedures are vital part of this task considering magnetic field presence [6, 7].

There are different IMRT QA verification solutions which work properly under magnetic field such as Sun Nuclear ArcCHECK-MR™ with helical diode array (Sun Nuclear Corporation, Melbourne, FL, USA), PTW Octavius™ 1500 with plane parallel ion chambers two-dimensional (2D) array (PTW GmbH Freiburg Germany), ScandiDos DELTA4 TM (ScandiDos AB, Uppsala, Sweden) with 2.5D diode array and Ashland Gafchromic™ EBT3 (Ashland Inc., Covington, KY, USA) film with 28 active layer. These systems can be compared in terms of dose response under magnetic field, detector type, number of detectors, measurement resolution, detector dimension, physical design, weight and cost.

Gafchromic™ EBT/EBT2 and EBT3 films were widely used for patient-specific dose verifications for long time [8-16]. EBT3 contains 28 active layer coated with 125 thickness matte polyester at the top and bottom of the film in order to stop Newton rings in contrast with EBT2. Both EBT2 and EBT3 offer very high spatial resolution with respect to diode and ion chamber (IC) systems [10-12]. Furthermore, there is no volume effect for small and micro beam fields and it generates minimum perturbation of the beam fluence with respect to IC arrays [8, 9]. In contrast with diode and IC arrays, poly-diacetylene monomer composite has nearly water equivalent atomic number (Z_eff _EBT3 = 7.46; Z_eff_ Water = 7.30) hence EBT3 does not have over-response with low-energy photons [10, 12, 13]. Finally, EBT3 gantry angle dependency is less and does not require any gantry angle inclinometer during irradiation [10,12]. On the other hand, film dosimetry has some disadvantages such as lot batch dependency, saturation time of film, light sensitivity of scanner, xenon lamp lifetime, landscape or portrait scan direction difference and lateral scanning effect [10-16].

As a consequence, physical characteristics, end-to-end (E2E) results, optical density (OD) consistency, dose distribution measurements in presence of tissue-air and tissue-lung interfaces with film and patient specific IMRT QA response of Gafchromic™ EBT2/3 films under magnetic field are interesting areas for research [17-22].

In this study, we investigated the clinical feasibility of Gafchromic™ EBT3 film using multichannel dosimetry with single scan method for patient specific IMRT QA under 0.35 T magnetic field.

## Materials and methods

Patient characteristics and treatment planning

Seventy patients treated with ViewRay MRIdian® Linac at our department between September 2018 and June 2019 were included in this study. Most common treatment locations were lung (26%), abdominal (40%) and pelvic (34%) regions. Either expirium or inspirium (17 sec or 25 sec MR scanning times) breath hold high-resolution (0.3 x 0.3 cm) MR images were acquired for lung and upper abdominal regions. Furthermore, free breathing (173 sec MR scanning time) with 0.15 x 0.3 cm high-resolution MR images were acquired for patients treated for lower abdominal and pelvic tumors.

Treatment planning computerized tomography (CT) images were acquired with Siemens Force 128 slice CT (Siemens Healthcare GmbH, Erlangen, Germany) using 0.15 cm slice thickness and similar MR simulation images were acquired in order to obtain electron density maps.

All step and shoot IMRT plans were optimized under magnetic field [17]. Monte Carlo (MC) dose engine was used with 0.1 or 0.3 cm calculation grids at ViewRay treatment planning system (TPS) version 5.2.5.14 with respect to field size. IMRT treatment plan metrics of the patients are shown in Table 1.

**Table 1:** Plan metrics for point dose and film QA. MLC: Multileaf collimator; MU: Monitor unit; GTV: Gross target volume; PTV: Planning target volume; QA: Quality assurance.

	# of Field	# of MLC	Plan MU	Beam on Time (min)	GTV Volume (cc)	PTV Volume (cc)
Range	[7 – 23]	[30 – 176]	[833 – 10759.5]	[1.39 – 17.93]	[0.34 – 330]	[1.21 – 421.24]
Mean			3256.5 2071.1	5.43 3.45	41.32 55.64	63.24 73.73
Median	21	59	2734.7	4.56	18.51	35.68

Prescription doses and fractionation schemes of each region are given in Table 2.

**Table 2:** Prescription doses and fractionation schemes of each region.

Prescribed Dose per Fraction (Gy)	# of Fraction	# of Patients	Region
7.25	5	16	Pelvic
6	5	1	Pelvic
3	20	1	Pelvic
2.5	28	1	Pelvic
20	3	1	Thorax
10	5	5	Thorax
8	7	1	Thorax
6	5	2	Thorax
4	15	1	Thorax
18	3	1	Abdominal
15	3	5	Abdominal
10	5	9	Abdominal
9	5	10	Abdominal
8	5	8	Abdominal
7	8	4	Abdominal
6	5	4	Abdominal

Prior to patient-specific QA evaluation with EBT3, basic scanning properties such as reflective or transmission scanning method, landscape or portrait scanning direction and scanning with or without glass cover were investigated but not published.

Film batch calibration

Four lots of EBT3 films with 20.32 x 25.40 cm^2^ sheet dimensions were used in order to perform 2D gamma analysis of the IMRT plans with multichannel dosimetry by single scan method [23,24]. One film sheet was cut into eight strips by taking into account film orientation with 2 x 20.32 cm^2^ dimensions for every lots separately. Calibration films were exposed to increasing dose values of 0 cGy, 50 cGy, 100 cGy, 200 cGy, 400 cGy, 600 cGy, 800 cGy and 1000 cGy under calibration reference conditions of SAD 90 cm at 5 cm depth inside the solid slab phantoms with 10 cm thickness under 0.35 T magnetic field. Ion chamber dose measurements were performed with PTW 31010 Semiflex 0.125 cc before calibration of film exposure procedure at the same depth in order to obtain consistent dose calibration of films [10, 12].

**Figure 1:**
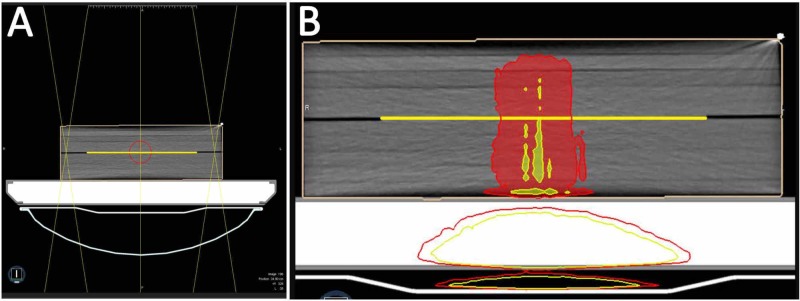
Film calibration irradiation setup for calibration curve. (A) Irradiation setup at 0 and 180 gantry angles in order to obtain homogenous dose map for calibration films. (B) Dose heterogeneity on the EBT3 film is at most 5%. Red and yellow with colorwash represent 100% and 105% relative doses with respect to prescription dose, respectively.

In contrast to 10 x 10 cm^2^ field size at gantry angle 0° irradiation set up, the calibration films were exposed at maximum field size (24.4 x 27.4 cm^2^) of multileaf collimator (MLC) with equally weighted two opposite fields at 0° and 180° gantry angles under 0.35 T magnetic field in order to avoid dose heterogeneity of 6MV FFF energy and create larger homogenous dose exposure throughout film pieces, as shown in Figure 1.

Calibration function was fitted onto the calibration dose points. It is not recommended to use polynomial fit function in the range of 0 cGy to 800 cGy, whereas reciprocal linear relation between color channel value and absorbed dose fitting function was employed in equation 1 instead of polynomial curve fitting [10, 12, 23, 24].

 (X(D) = a+(b/(D-c)) (1)

where X is red, green and blue optical density, a, b and c are curve fitting coefficients and D is the dose in Gy. In addition, transmission mode with no color correction of positive film scanning was employed for all EBT3 film scans. We always used the same film scanning orientation during scanning [10,12]. Moreover, 72 dpi image resolution with 48-bit red, green and blue (RGB) multichannel was used 24 hours after irradiation in order to reach full saturation of the polymerization process [10, 12, 23, 24].

Gafchromic™ EBT3 film dose calculation and measurement with single scan method

A 20.32 x 25.40 cm^2^ sheet size Gafchromic™ EBT3 film was placed between 10 cm thickness slab phantoms at a depth of 5 cm and scanned with CT. Our intent to acquire a CT scan is to asses electron density map of phantom and to obtain accurate dose calculation.

IMRT plans were projected to the phantom with the same gantry angles and plan isocenter was placed to center of EBT3 film. The recommended dose analysis for multichannel dosimetry ranges between 20 cGy and 800 cGy for EBT3 film [11, 12]. The EBT3 films optical density (OD) saturated at the dose of 800 cGy and more. Thus, IMRT dose prescriptions higher than 800 cGy were rescaled to less than 800 cGy due to the saturation concern of the film.

In order to create accurate and reproducible film dosimetry for IMRT QA, single scan method with multichannel dosimetry was applied for all EBT3 film QA irradiation procedures. Firstly, film sheets were cut into three strips with the consideration of same orientation of these strips and calibration films. One strip was used for film QA, second strip was unexposed for background dose and third strip was exposed for reference dose [24].

The exposed reference film strip was used to re-scale the calibration curve function to fit the responses of that specific scan for each film lot. This eliminates the scan-to-scan variability from all sources and enables reliable absolute dosimetry [24].

Multichannel scanning method

The Epson 12000 XL scanner (Seiko Epson Corporation, Nagano, Japan) was used in this study to scan calibration and QA films. Epson flatbed scanners consist of xenon cold light lamp. Therefore, scanner was warmed up at standby position and four consecutive preview scans of whole active scanning area were performed in order to trim light lumen homogeneity before acquiring EBT3 film scans. Lateral nonuniformity effect of flatbed scanners are well known for long time [10, 12, 23, 24]. Perpendicular or parallel alignment of the film to the scan direction effects the pixel values of OD. Hence, OD deviation distribution of scanner was mapped for both landscape and portrait direction. Least effected region from lateral nonuniformity was determined and films were located at this axis before films were scanned.

It is well known that curling of films during scanning negatively affects 2D absolute dose analysis [25]. Films can be easily flattened by using a glass material which covers the active acquiring area during scanning. In this study, all scanning procedure was performed by using, covering the film by glass.

Gamma analysis of IMRT QA with multichannel dosimetry

FilmQA Pro 2016 (Ashland Inc., Covington, KY, USA) with RGB multichannel dosimetry was used for each lot of EBT3 film calibration curve and patient IMRT QA. We have evaluated, calculated and measured absolute dose maps by using gamma analysis method with dose difference % (DD) and distance to agreement (DTA) criteria [23-25].

RGB channel images were employed to establish dose, uniformity and consistency maps from 48-bit scanned films [23, 24]. This methodology improved gamma analysis evaluation for film dosimetry.

EBT3 dose map and coronal dose plan were matched according to laser markers on the EBT3 film. The matching was achieved with translational and rotational fusion.

Local Gamma analysis evaluation was performed for RGB channels particularly for both 2% DD/2 mm DTA and 3% DD/3 mm DTA criteria with 0 cGy threshold. The region of interest of gamma analysis comparison was evaluated by adding extra 3 cm for x and y directions to irradiated area outline of exposed film. More than 95% of passing rate was accepted as a successful QA for local gamma comparison between TPS dose calculations and film dosimetry measurements. Passing rates between 90% and 95% were defined as acceptable. Less than 90% of passing rate was accepted as failure of QA.

## Results

Gamma analysis

**Figure 2:**
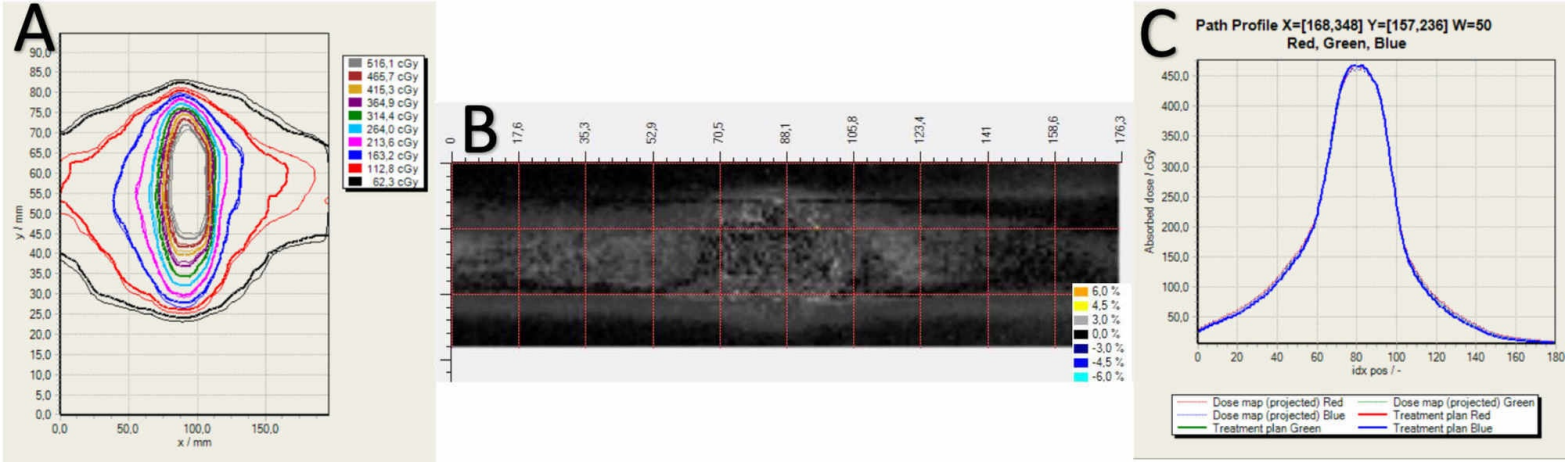
Multichannel film dosimetry with single scan method of gamma analysis with 2% DD/2 mm distance to agreement (DTA). (A) Isodose comparison of film and treatment planning system (TPS) dose plane. Bold and light isodose lines represent film and TPS maps, respectively. (B) Gamma evaluation map of red channel for 2% /2 mm. (C) 20-pixel averaged profile lines across the comparison map for three channels.

Figure 2 represents a typical multichannel gamma analysis of patient QA irradiated with EBT3 film with single scan method.

**Figure 3:**
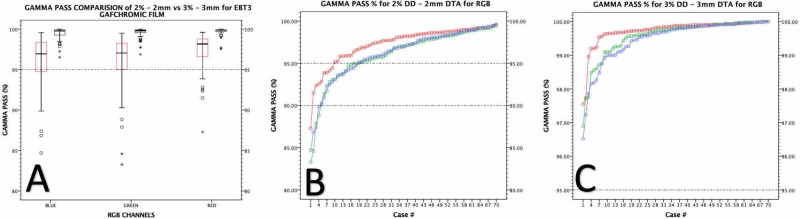
Gamma passing rates of red, green and blue channels. (A) Merged plot of 2% DD / 2 mm DTA and 3% DD / 3 mm distance to agreement (DTA) gamma evaluation criteria of passing rates. (B) Gamma passing rates of red, green and blue channels for 2% / 2 mm. (C) Gamma passing rates of red, green and blue channels for 3% / 3 mm.

The Whisker box and scatter plots of gamma evaluation results of 70 cases for both 2% / 2 mm and 3% / 3 mm criteria in terms of triple channel passing rates are shown in Figure 3.

The mean gamma passing rate is found to be 97.3% ± 2.26%, 96.0% ± 3.27% and 96.2% ± 3.14% for RGB channels, respectively in terms of 2% / 2 mm. The median passing rate is found to be 98.2%, 97.1% and 96.95% for RGB channels, respectively, in terms of 2% / 2 mm (Table 3).

**Table 3:** Gamma passing rate result statistics of 70 patients treated with IMRT for 2% DD / 2 mm DTA and 3% DD / 3 mm DTA evaluation parameters. Red, green and blue refer to channels of the film dosimetry. IMRT: Intensity modulated radiotherapy; DD: Dose difference; DTA: Distance to agreement.

Gamma Passing Rate (%)	2% DD / 2 mm DTA			3% DD / 3 mm DTA		
	Red	Green	Blue	Red	Green	Blue
Mean	97.3 ± 2.26	96.0 ± 3.27	96.2 ± 3.14	99.7 ± 0.41	99.6 ± 0.59	99.5 ± 0.67
Median	98.2	97.1	96.9	99.9	99.8	99.8
Range	87.3 - 100	83.2 - 100	84.7 - 100	97.5 - 100	96.9 - 100	96.5 - 100

The 91% of the QA analysis was found to be more than 95% passing rate with success. The 99% of QA analysis was >90% for 2% / 2 mm. The minimum gamma passing rate is found to be 87.3%, 83.2% and 84.7% for RGB channels, respectively.

The mean gamma passing rate is 99.7% ± 0.41%, 99.6% ± 0.59% and 99.5% ± 0.67% for RGB channels, respectively in terms of 3% / 3 mm. The median passing rate is 99.9%, 99.8% and 99.8% for RGB channels, respectively in terms of 3% / 3 mm. The 100% of QA analysis was more than 95% passing rate for 3% / 3 mm. The minimum gamma passing rate is 97.5%, 96.9% and 96.5% for RGB channels, respectively.

## Discussion

MRIGRT is a new robust treatment method. QA is an integral part of safe implementation of IMRT treatments under MRI guidance [6, 7]. In this study, we have evaluated the feasibility of Gafchromic™ EBT3 film for patient specific QA using gamma analysis with multichannel film dosimetry under 0.35 T magnetic field in 70 cases.

There are couple of studies in the literature on patient specific QA under 0.35 T magnetic field. Firstly, Li et al. reported their QA findings in 34 patients. They used EDR2 film dosimetry under 0.35 T in 30 patients [7]. They reported mean gamma passing rate as 94.6% (ranging from 87.4% to 100%) for 3% / 3 mm. In our study, 100% of QA gamma analysis was over 95% passing rate for 3% / 3 mm with mean 99.7% ± 0.41% (ranging from 97.5% to 100%). Moreover, there is only one unacceptable QA finding among one out of 70 patients for 2% / 2 mm. The 91% and 99% of the all QA analysis was over 95% and 90% with mean passing rate 97.3% ± 2.26% ranging from 87.3% to 100%, respectively for 2% / 2 mm. Our results are superior for 2% / 2 mm and 3% / 3 mm analysis in concordance with author’s results. The possible explanation of this difference is most probably due to use of EDR2 with single red channel analysis in their study. Same investigators also performed gamma analysis using 3D ArcCHECK-MR™ and they found that their mean passing rate was 98.9% ± 1.1% (ranging from 95.8% to 100%) using relative 3% / 3 mm gamma criteria. Their 2% / 2 mm gamma criteria passing rate results were not reported in their manuscript by the authors [7].

Secondly, Ellefson et al. reported their average gamma passing rates in 19 patients as 96.9% ± 6.8% and 84.0% ± 17.5% for 3% / 3 mm and 2% / 2 mm, respectively using ArcCHECK-MR™ [17]. They concluded that their average gamma passing rate results for 3% / 3 mm was acceptable. On the other hand, they mentioned that the passing rate results for 2% / 2 mm were lower and found to be unacceptable. The main reason for this discrepancy can be due to combined angular and dependencies up to 10% and corrected by using virtual inclinometer at ArcCHECK-MR™ QA system [26, 27]. However, this correction cannot be used due to incompatibility of the virtual inclinometer with ViewRay’s multiple beams [17].

The EBT3 film dosimetry has several advantages such as being water equivalent, easy to use, no need for correction factors, structural flexibility, isolated for water and moisture penetration, no dependency with irradiation angle and high resolution of detection with 28 active layer thickness. However, film dosimetry has some disadvantages such as inconsistency at radiochromic film characteristics, saturation time, lot batch dependency, light sensitivity, landscape and portrait scan dependency, peripheral scanner devices limitations such as xenon lamp lifetime, scanning region calibration, lateral effect and uncertainties of film dosimetry [10, 12, 13, 23, 24, 28-30]. The main sources of uncertainties were defined by Bouchard et al.: film manufacturing or film homogeneity, film manipulation (i.e., storage, cutting), film irradiation, film digitalization and film response characterization with absorbed dose [29]. On the other hand, Sorriaux et al. denoted a total uncertainty below 1% in the radiotherapy dose range (>1.5 Gy) in photon mode using EBT3 films [30]. In our study, no attempt was made to correct for the uncertainties involved in film dosimetry. In addition to all these, OD evaluation of radiochromic film under magnetic field needs more study. There are several researches on that topic [18-22].

Reyhan et al. reported dose perturbations using EBT2 Gafchromic™ films under 1.5 T magnetic field [18]. Their measurements revealed 4% dose deviations on average under magnetic field, compared to without the presence of MRI. It was also concluded that accurate dosimetry could be obtained by applying a correction factor to the red-channel pixel value prior to dose conversion [18]. Furthermore, Reynoso et al. investigated the effects of magneto-kinetic changes on crystal orientation and polymerization within the active layer of EBT2 film [19]. Under the presence of 0.35 T, net OD decreased by an average of 8.7%, 8.0%, and 4.3% in the red, green, and blue channels, respectively. The dose differed by up to 15% at 17.6 Gy for their findings.

Both Reyhan et al. and Reynoso et al. had investigated film characteristics of EBT2 with the presence of 1.5 T or 0.35 T magnetic field prior to EBT2 was superseded by EBT3 from manufacturer. The possible reason for their findings can be explained with asymmetric clear polyester coated layer design of EBT2. On the contrary, EBT3 active layer is coated with symmetric 125 matte polyesters [10, 12]. Therefore, EBT3 film seems to be more suitable than EBT2 under presence of magnetic field [18-22]. Cusumano et al. experimentally studied estimating the impact of a 0.35 T transverse magnetic field on dose distribution in presence of tissue-air and tissue-lung interfaces [21]. Their investigation consisted of comparing experimental measurements performed in presence of magnetic field by radiochromic film dosimetry, to Monte Carlo simulations, which were performed with the presence and the absence of magnetic field. Their experimental measurements were realised using Gafchromic EBT3 films with novel sum signal method [21].

Delfs et al. used EBT3 films for measurement of response of 6 MV photons under magnetic fields in the literature [20]. They found small dose uncertainties under the magnetic fields at the rate of -2.1%, whereas relative dose distribution remained acceptable. Similarly, Barten et al. found a small deviation of less than 1.5% in EBT3 dose response after irradiation under 0.35 T using radiofrequency energy at on and off mode [22]. Findings of Delfs et al. and Barten et al. were within the same deviation line. Moreover, Barten et al. stated that EBT3 film was useful for absolute dosimetry during real-time MR imaging independent of its orientation in the B0 magnetic field and a suitable dosimeter for patient-specific QA measurements with 0.35 T MRI-radiotherapy devices.

As a result, the findings of our study support the findings of the authors who studied EBT3 film characteristics under 0.35 T magnetic field and also our results show that signal scan method with multichannel analysis achieved reliable results under low tesla magnetic field [20-22].

## Conclusions

Gafchromic™ EBT3 film dosimetry is consistent QA tool for gamma analysis evaluation of the treatment plans even with 2% DD / 2 mm DTA under 0.35 T magnetic field presence. Our results support that IMRT QA with multichannel dosimetry is a suitable tool for implementation of safe MR-guided treatments using ViewRay MRIdian® Linac.
